# Determination of the density of human nuclear cataract lenses

**DOI:** 10.3892/mmr.2013.1673

**Published:** 2013-09-09

**Authors:** KE XU, YANSHENG HAO

**Affiliations:** Department of Ophthalmology, Peking University Third Hospital, Haidian, Beijing 100191, P.R. China

**Keywords:** nuclear cataract, lens density, Scheimpflug photography

## Abstract

The aim of the present study was to detect senile nuclear cataract lens density and provide a quantitative measurement of lens density for the long-term clinical observation of cataracts. An Anterior Segment Analysis System was used to detect the lens density of 422 simple senile cataract eyes and normal contralateral eyes. The density values were taken at the optical axis at various depths measured from the anterior capsule. The differences in lens density at various photographic orientations and in various nuclear grading groups were investigated. The density at 90º orientation was larger than that at 30 and 150º (P<0.05). The density was reduced beyond the 1.0-mm depth point in all the lenses. Prior to the 2.5-mm depth point, there were differences among the five grade groups (P<0.05). Beyond the 3.0-mm depth point, values in all the grade groups were low and not significantly different (P>0.05). In the grade 4 group, the decrease was more evident. The average density values at all the depth points showed a tendency to increase with increasing nuclear grade. Measuring the density of the anterior half of the lens at 90º orientation is therefore more reliable compared with that of the posterior half and at other orientations. Results of the present study have shown that the Anterior Segment Analysis System may be used to detect senile nuclear cataract lens density and provide a quantitative measurement of lens density for long-term clinical observation of cataracts.

## Introduction

Cataracts have proven to be a difficult entity for clinical study, partly due to the difficulty in developing an objective, accurate and reproducible lens evaluation system. Evaluation systems based on visual acuity fail to detect early lens changes and are confounded by the presence of other ocular abnormalities. Evaluation systems based on clinical impressions often have large inter- and intra-observer variability. Optical photography, however, has small variability and is more objective than other methods (1–4). One of the advantages of Scheimpflug photography is the large depth of field resulting in an extremely clear image. Since 1966, Scheimpflug photography has been used for the documentation and measurement of the anterior eye segment (5). The first prototypes constructed by Brown were never produced as commercial versions (6). An SL-45 camera using film as data-recording medium was manufactured in 1979 by Topcon Optical Company (Japan) and for several years it was the only Scheimpflug camera available (3). In 1984, Zeiss introduced the SLC system, which was the first video-based Scheimpflug camera (7). Apart from these independent camera systems, Sasaki *et al*(8) designed a photographic unit that may be attached to the Topcon SL-6E slit lamp microscope. Rapid progression in digital imaging and computerized image analysis stimulated the development of Scheimpflug cameras, including the Oxford Modular Cataract Image Analysis System (case 2000) and the Nidek Eye Analysis System 1000 (9,10). In 1996, the photographic camera part of the Topcon SL-45B (which was also the first rotating Scheimpflug camera) was replaced with a CCD camera unit, enabling direct image acquisition and computer-assisted analyses (11).

**Figure 1:**
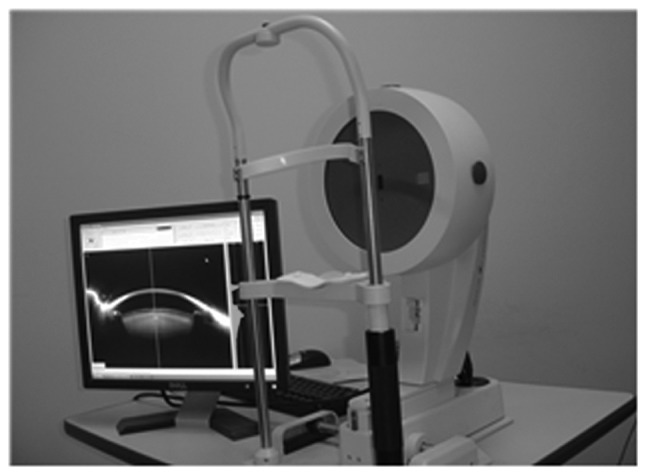
Scheimpflug Imaging System.

Pentacam is a newly developed anterior eye segment analysis system (Oculus Company, Wetzlar, Germany; Fig. 1) and uses the Scheimpflug imaging principle. It uses a diode with an ultraviolet-free blue light with a wavelength of 475 nm as the light source and analyzes lens density through a reflecting image. The system has 25,000 measuring points and thus, the image is extremely clear. Moreover, it performs accurate anterior segment analysis, including anterior chamber depth, anterior chamber angle and anterior chamber volume. It also performs anterior eye segment three-dimensional model rebuilding.

In the present study, this system was used to detect senile nuclear cataract lens density, with the purpose of providing a quantitative measurement of lens density for the long-term clinical observation of cataracts.

## Patients and methods

### Patients

Between January and June 2006, ophthalmic examinations were performed on outpatients with cataracts at the Peking University Eye Center (Beijing, China). Subjects with corneal opacity, glaucoma, eye trauma and other eye diseases, including diabetic retinopathy, were excluded. Informed consent was obtained from all the patients. The study was approved by the Ethics Committee of Peking University Third Hospital.

### Imaging

**Figure 2:**
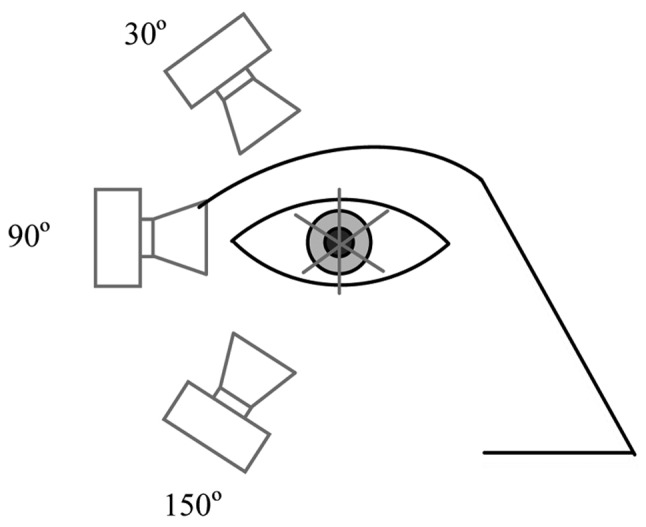
Slit lamp Scheimpflug images of the lens were captured at three orientations to obtain 30, 90 and 150º meridional section images.

Simple nuclear cataract eyes and transparent eyes from these patients were imaged by color slit lamp photography (Zeiss, Yena, Germany) and Scheimpflug photography was performed with the Anterior Segment Analysis System (Scheimpflug Imaging System; Pentacam, Oculus Company, Wetzlar, Germany) through pupils maximally dilated with tripicamide ophthalmic compound (0.5% tropicamide and 0.5% phenylephrine) by one technician. Color slit lamp images of the lenses were captured from the temporal side with the beam at a 45º angle with fixed width and length of the slit. Slit images of the lenses were obtained from the temporal side (12) at three orientations (30, 90 and 150º) and meridional section images of each lens were obtained (Fig. 2) (13). If blockage of the eyelid was marked, adhesive tape was used to lift the eyelids in order to reduce the blockage. Of 238 patients with a mean age of ~70 years (range, 40–94 years), 422 eyes were evaluated. There were 234 male and 188 female eyes.

**Figure 3:**
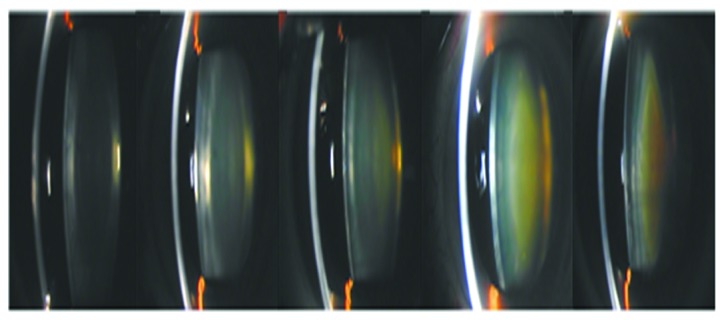
Lens Opacities Classification System II. Nuclear grades 0, 1, 2, 3 and 4 are shown from left to right.

**Figure 4:**
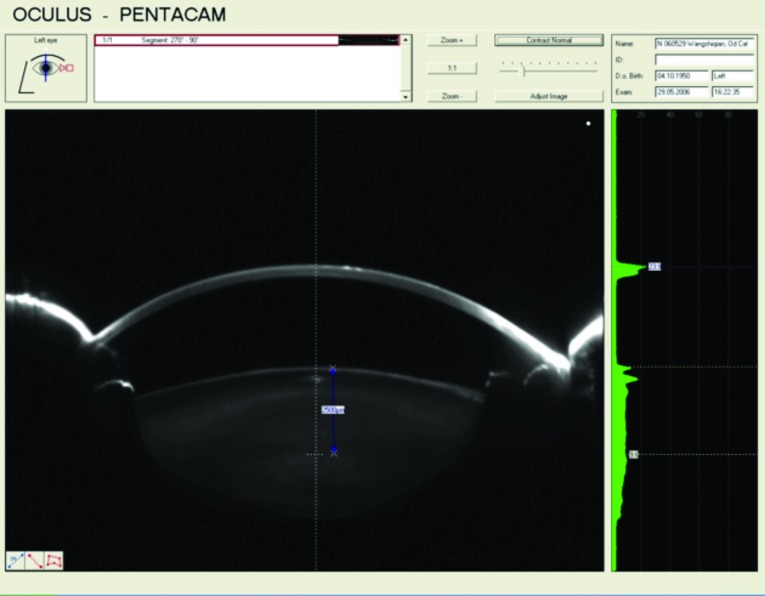
Obtaining lens density values in the Scheimpflug image. The optical axis, shown as a white dotted line, was provided by the program automatically. Using the scale (blue line) provided by the program, the distance between any two points may be marked. The density values corresponding to the optical axis are shown in the green graph on the right. In this example, the depth was 2.5 mm and the density value was 9.6 units.

Patients were examined and nuclear hardness was graded by a doctor using the Emery-Little classification system (Fig. 3). Another doctor obtained the lens density values without knowledge of the grading of nuclear hardness. The values were taken at the optical axis at various depths measured from the anterior capsule. The optical axis was determined automatically by the software (Pentacam lens densitometry program; Oculus, Wetzlar, Germany). In total, eight depth measurements were taken at 0.5-mm intervals using pointers. Notably, interference of the reflection of the Scheimpflug photography system light source was not completely avoided when obtaining the density values; thus, if the depth point fell on the reflection of the light source the value was dismissed (Fig. 4).

### Statistical analysis

A paired t-test was applied to test the significance of differences in the three orientation groups and was repeated at each depth point. One-way analysis of variance was applied to test the significance of differences among five grading groups at each depth point of the optical axis. Statistical analyses were performed with SPSS 11.0 statistical software (SPSS, Inc., Chicago, IL, USA). P<0.05 was considered to indicate a statistically significant difference.

## Results

### Mean density of the three orientation groups at various depths of the optical axis

**Figure 5:**
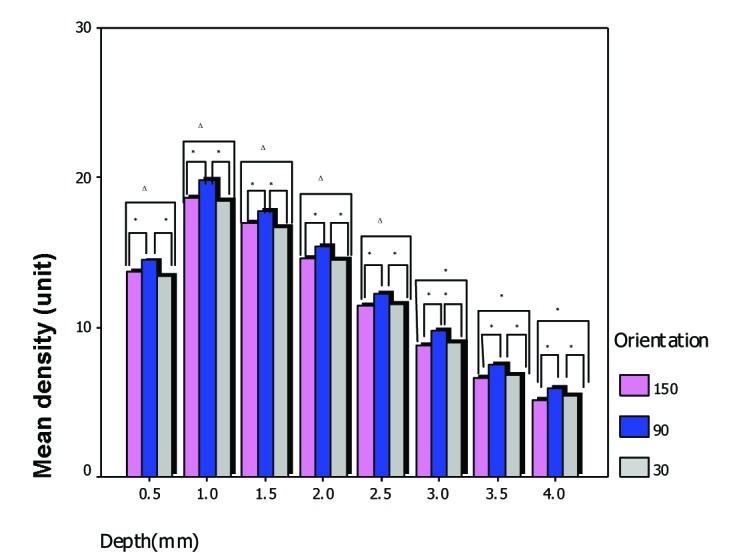
Mean density of the three orientation groups at various depths of the optical axis. At 90º, the mean density value was slightly larger (P<0.05). No significant difference in density value was noted between the 30 and 150º groups prior to the 3-mm depth point. ^*^P<0.05, ^Δ^P>0.05.

All the groups showed almost the same density value at each depth point. The number of cases examined and the mean density of the three orientation groups at each depth point of the optical axis are shown in Table I. There was a statistically significant difference between the 90º group and the two other orientation groups (both P<0.05). At 90º, the mean density value was larger. No significant difference in density value was noted between the 30 and 150º groups prior to the 3-mm depth point and the three groups showed a decline in density beyond the 1.0-mm depth point (Fig. 5).

### Mean density of the five grades at each depth point of the optical axis at 90º

**Figure 6:**
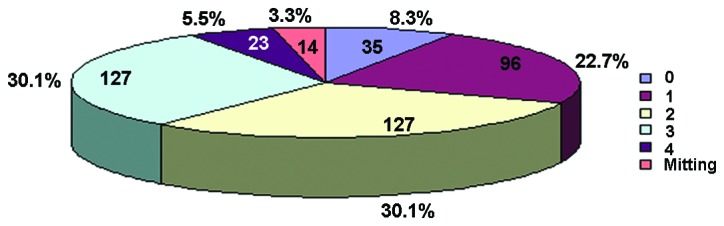
Distribution of grading in 422 eyes.

Of 422 eyes, there were 35 with grade 0, 96 with grade 1, 127 with grade 2, 127 with grade 3 and 23 with grade 4; in 14, the value was not determined due to loss of color in the slit lamp photographs (Fig. 6). The mean densities of the various grades are shown in Table II.

### Mean density of five grade groups at various depths of the optical axis at 90º (depth and nuclear grade)

**Figure 7:**
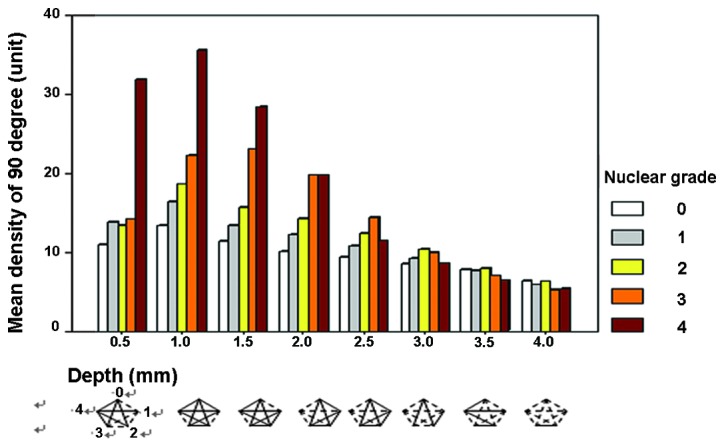
Mean density of five grade groups at various depths of the optical axis at 90º (depth is x-axis). The pentacles showed if there was a significant difference between each other in five grading groups. The solid line indicates a significant difference. The dotted line indicates there was no significant difference.

**Figure 8:**
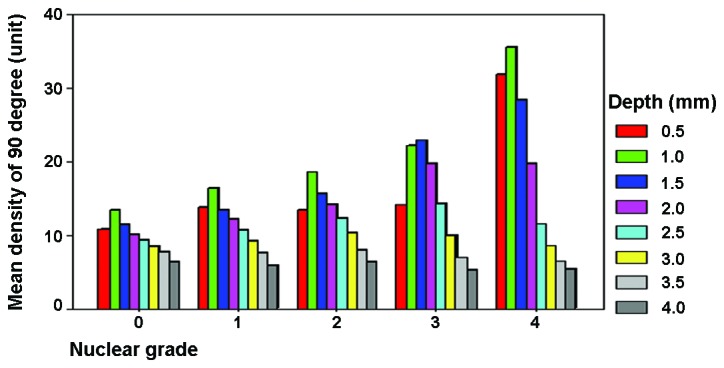
Mean density of the five grade groups at various depths of the optical axis at 90º (nuclear grade is x-axis). All the nuclear grade groups showed a decline in density beyond the 1.0-mm depth point. In the grade 0 group, the decrease was slow and the tendency was greater and more obvious as the grade increased.

Differences in density of the five grade groups at each depth point of the optical axis at 90º are shown in Figs. 7 and 8. At the 0.5-mm depth point, there were no significant differences in density among grades 1, 2 and 3 (all P>0.05). However, there was a significant difference between grade 0 and the other groups, as well as between grade 4 and the other groups (P<0.05). These data suggest that in grade 4 lenses, the density is increased in the superficial part of the lens, however, the differences among grades 1, 2 and 3 were not obvious.

There were significant differences among almost all grades at the 1.0- and 1.5-mm depth points (all P<0.05) and the mean density showed a tendency to increase with increasing nuclear grade.

There were significant differences among grades 0, 1, 2 and 3 at the 2.0- and 2.5-mm depth points (P<0.05) and the mean density showed a tendency to increase with increasing nuclear grade. However, no significant differences were noted between grade 4 and the other grades (P>0.05).

Beyond the 3.0-mm depth point, values in all the grade groups were low and no significant differences were noted (all P>0.05).

## Discussion

Previous studies have been performed to show the correlation between the lens peak and mean density and clinical nuclear grade (1), as well as lens density and age (2,12), and follow-up observation of lens densitometry (14). Results of this study demonstrated that the standard deviation of density values of three orientation groups at various depths of the optical axis was large. One explanation is that each orientation group included all nuclear grades, thus the range of the values was large. Of the density values of the five grade groups at various depths, the standard deviation of grade 4 was large and this was likely due to the small number of cases. Important factors that affect the range of values are the positioning of the patient's head and the fixation of the examined eye. The blockage of eyelids and eyelashes also affects the range of values. In addition, the thickness of lens is different; however, values were taken from the optical axis at various depths measured from the anterior capsule. With the Scheimpflug Anterior Segment Analysis System, a reflection of the system light source near the anterior capsule was consistently found when the density value was abnormally large. This affected the accuracy of determination of the mean density and peak density of the whole lens. In order to be as accurate as possible, if the depth point fell on the light source reflection, this value was dismissed.

The current data indicated that the 90º orientation values were the most reliable, which is possibly due to minimal blockage by the eyelids and eyelashes. Thus, the values of the 90º group were used for the statistical analysis.

The thickness of the lens is 4–5 mm. In theory, the density should distribute symmetrically from the center. In this study, all nuclear grades showed a reduction in density beyond the 1.0-mm depth point and this tendency was more obvious as the grade increased. An important reason for this observation is that the Scheimpflug analysis system analyzes the lens density through a reflected image. A high level of back-scatter light in the anterior nucleus decreases the amount of light transmitted to the posterior parts of the nucleus, thus, making them appear less dense. In addition, heavily pigmented nuclear cataracts may absorb a large amount of back-scattered light and thus, appear less dense. In practical terms, the anterior part of the lens is the least affected by any of these changes and is the easiest to analyze (15). The results also showed that beyond the 3.0-mm depth point, values in all the grade groups were low, without significant differences.

Semiautomatic camera alignment and focusing is one advantage of the anterior eye segment analysis system used in the present study. It is a highly automated method of photography and analysis that is simple to operate without extensive operator experience. The 180º autorotation of the system captures high-quality images of the whole anterior eye segment and the software determines the lens density at any point in any meridional section. Important factors that affect reproducibility of the data includes the positioning of the patient's head, fixation of the examined eye and blockage by the eyelid and eyelashes. As the measuring depth increased, the effect of the physics of optics was unavoidable, which affected the reliability of the values.

In conclusion, the Anterior Segment Analysis System may be used to detect senile nuclear cataract lens density and provide a quantitative measurement of lens density for long-term clinical observation of cataracts.

## Figures and Tables

**Table I:** Mean density of the three orientation groups at each depth point of the optical axis.

	150º			90º			30º		
			
Depth, mm	n	Mean	SD	n	Mean	SD	n	Mean	SD
0.5	412	13.712	9.2856	411	14.460	10.5877	405	13.439	9.3138
1.0	408	18.621	8.3560	403	19.800	9.8208	408	18.465	8.5854
1.5	414	16.964	7.5371	412	17.770	8.2408	412	16.720	6.007
2.0	421	14.564	6.0807	421	15.410	7.1814	417	14.483	5.9297
2.5	421	11.445	4.3968	422	12.277	4.5001	421	11.570	4.2786
3.0	421	8.815	3.1971	422	9.730	3.2123	421	8.994	3.0838
3.5	421	6.592	2.2155	422	7.543	2.2943	421	6.856	2.2014
4.0	421	5.177	1.5762	422	5.909	2.0259	421	5.442	2.0488

SD, standard deviation.

**Table II:** Mean density of the five grades at each depth point of the optical axis at 90º.

	0.5 mm		1.0 mm		1.5 mm		2.0 mm		2.5 mm		3.0 mm		3.5 mm		4.0 mm	
								
Grade	Mean	SD	Mean	SD	Mean	SD	Mean	SD	Mean	SD	Mean	SD	Mean	SD	Mean	SD
0	10.960	3.1226	13.397	4.1579	11.457	3.4342	10.146	2.8129	9.320	2.3337	8.526	2.0535	7.809	1.7255	6.509	3.4418
1	13.878	9.8328	16.451	6.4321	13.482	4.0230	12.278	3.3263	10.827	2.7978	9.221	2.2743	7.604	1.9366	5.886	1.7248
2	13.399	6.8027	18.642	5.3914	15.696	4.0446	14.224	3.6382	12.343	3.2979	10.376	3.0379	8.079	2.2737	6.413	1.9247
3	14.148	10.3247	22.246	9.5370	22.961	8.6140	19.763	7.4675	14.325	5.5429	10.029	3.6553	7.056	2.2554	5.328	1.3853
4	31.848	22.6109	35.513	22.0768	28.396	15.3646	19.774	17.4216	11.535	7.4943	8.626	5.2924	6.526	3.8077	5.391	2.9915

SD, standard deviation.
